# Advancements in global water and sanitation access (2000–2020)

**DOI:** 10.1038/s41598-025-90980-7

**Published:** 2025-02-21

**Authors:** Hiroki Ando, Masaaki Kitajima, Taikan Oki, Michio Murakami

**Affiliations:** 1 Division of Environmental Engineering, Faculty of Engineering, Hokkaido University, North 13 West 8, Kita-ku, Sapporo, Hokkaido 060-8628 Japan; 2 Mel and Enid Zuckerman College of Public Health, University of Arizona, Tucson, AZ 85724 USA; 3 Research Center for Water Environment Technology, Graduate School of Engineering, The University of Tokyo, 2-11-16 Yayoi, Bunkyo, Tokyo 113-0032 Japan; 4 Department of Civil Engineering, Graduate School of Engineering, The University of Tokyo, 7- 3-1 Hongo, Bunkyo, Tokyo 113-8656 Japan; 5 Center for Infectious Disease Education and Research, Osaka University, 2-8 Yamadaoka, Suita, Osaka 565-0871 Japan

**Keywords:** Environmental sciences, Environmental social sciences

## Abstract

Globally equitable access to safely managed drinking water and sanitation is one of the major aims of the United Nations’ Sustainable Development Goals, specifically SDG goal 6. We assessed global-scale progress toward this goal from 2000 to 2020 with access rates to improved drinking water and sanitation services that are adjusted for socioeconomic, political, and hydrological conditions. We found that the adjusted access rates in 2020 were lower than 2000, although not-adjusted access rates had increased. Access rates improved more slowly in higher-GDP countries than in lower-GDP ones during this period. These show that access rates in lower-income countries improved, but were still lower, compared with those in countries with the same social conditions in 2000. This suggests the recent progress toward this goal has not aligned well with social development, emphasizing need for reflecting national management, international cooperation, and investment in water-related infrastructures to achieve the goal by 2030.

**Supplementary Information:**

The online version contains supplementary material available at 10.1038/s41598-025-90980-7.

## Introduction

Drinking water, sanitation, and hygiene (WASH) are fundamental components for human life and society. The lack of accessibility to WASH services has been linked with the prevalence of diseases^1,2^, low national economic productivity^3^, and gender inequality^4^. Globally, the proportion and absolute number of people with access to improved WASH services has increased over time; however, access inequality remains rampant^5,6^and, as of 2022, at least 700 million people were living under unimproved WASH levels^4^. Even in the 21st century, about 1.4 million annual deaths could have been prevented with safely managed WASH services^1,2^.

To attain both adequate and equitable access to safely managed WASH services on a global scale, international organizations have set middle-term goals that include targets and indicators, which have been revised over time^7^. In 2001, United Nations Millennium Development Goal (MDG) Target 7.C emerged, but the indicators used for monitoring the progress of the MDGs were too simple to reflect water quality and the accessibility of these services accurately^7–10^. In 2015, the MDGs were reorganized as Sustainable Development Goals (SDGs), which included Targets 6.1 and 6.2, aiming to achieve adequate and equitable access to WASH services^11^. This update led to improved indicators that account for water quality and accessibility. Water collection time and water quality were also incorporated to determine WASH service levels in the SDG framework, although service levels were considered only in terms of the water source category, such as pipe water, as noted in the MDG framework^7^. Progress toward the targets has been monitored by the WHO/UNICEF Joint Monitoring Program for Water Supply, Sanitation and Hygiene (JMP), based on specific indicators^12^.

Both access rates and progress have been investigated from multiple perspectives to provide helpful insights for achieving SDG targets. To date, economic capabilities, such as per capita GDP, have been identified as a factor associated with the proportion of a given population receiving improved WASH services^7,13,14^. In addition, previous studies have reported an association between equitable access to safely managed services and water quality with the social and political dimensions of water^5,15,16^. However, despite these being potential associated factors, progress has mainly been discussed based on increased numbers and proportions of people with access to improved WASH services^4^. This indicator is useful for understanding the magnitude of progress, but it does not allow for an evaluation of progress itself, as a function of socioeconomic and political factors.

In the present study, we compared access rates for drinking water and sanitation between 2000 and 2020 with a consideration of socioeconomic, political, and hydrological conditions, aiming to provide insights into past progress toward universal access on a global scale. The association of access to WASH services with socioeconomic and political conditions has already been established^5,7,13–16^. Hydrological factors, such as per capita renewable internal freshwater resources, are expected to directly affect available water resource and likelihood of people’s access to WASH services. We identified an association between the access rate and these factors and then compared access rates for drinking water and sanitation between 2000 and 2020 by adjusting for levels of socioeconomic conditions. We also analyzed the relationship between improved access rates in the past two decades and social and hydrological conditions, to identify potential factors prompting changes in these rates.

## Main (results)

### Summary of JMP data

We extracted data from the JMP website on access rates by country between 2000 and 2020^12^. We chose this timeframe because the latest socioeconomic data were only available through 2020 at the time of this writing. Although indicators used during MDG era did not account for water quality and the accessibility of these services^7–10^, the JMP provides data on access rate to each service level of drinking water and sanitation since 2000: safely managed service, basic service, at least basic service, limited service, unimproved service, and surface water (only for drinking water) or open defecation (only for sanitation)^4^. JMP data have primarily been collected through household surveys and censuses in regions accessible to researchers, which may result in biased data. To address the problem, WHO has collaborated with UNICEF to enhance monitoring system of WASH service^17^. In this study, we performed analysis based on the assumption that the presented JMP data reflect the national status of access rates well.

**Fig. 1:**
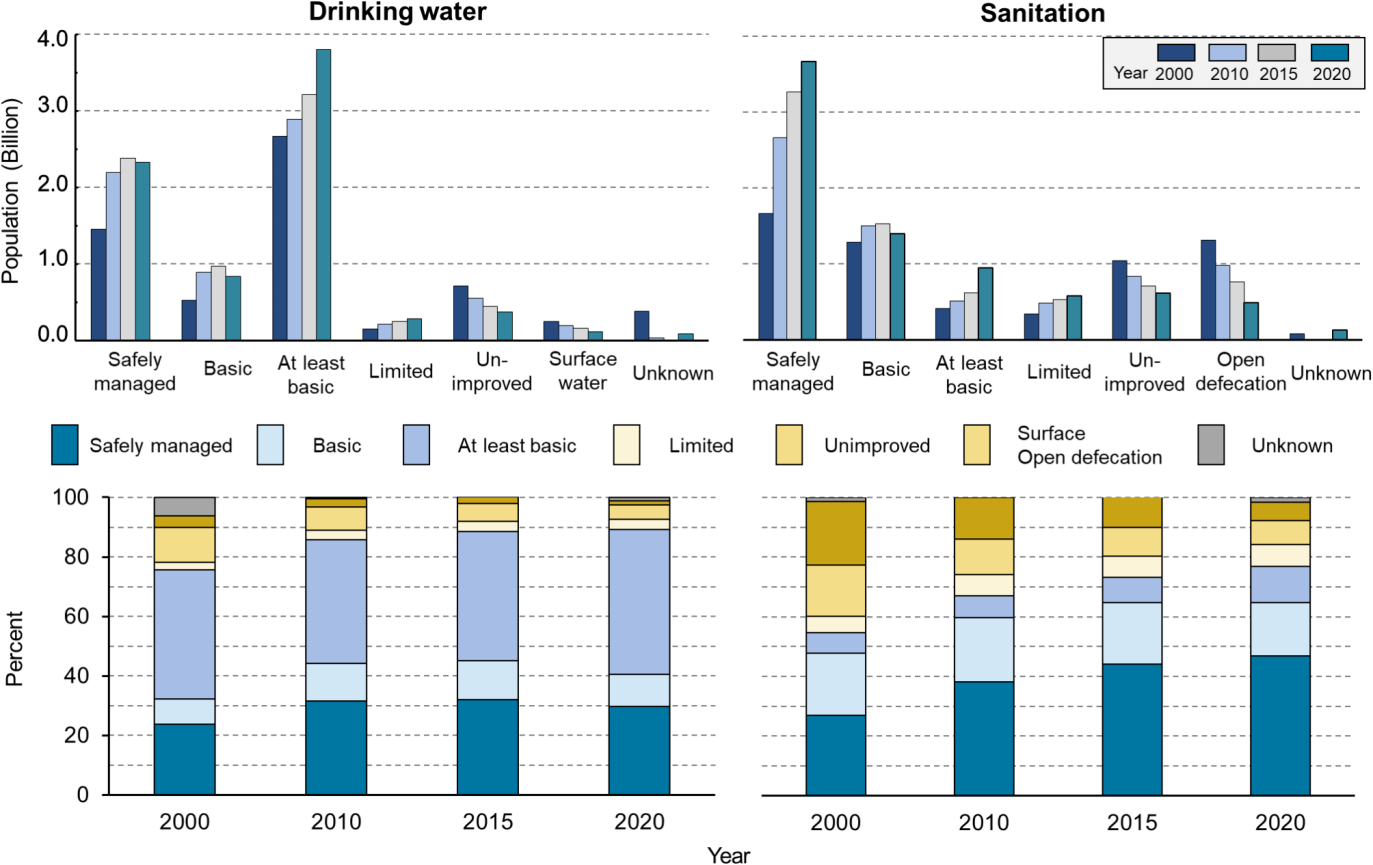
The number (upper panel) and percentage (lower panel) of the population with access to drinking water and sanitation. The total population in the globe including countries without access rate data of WHO/UNICEF Joint Monitoring Program for Water Supply, Sanitation and Hygiene (JMP) were retrieved from the World Bank^18^.

The JMP data show a steady increase in both the absolute number and percentage of the population with access to safely managed or basic services for drinking water and sanitation between 2000 and 2020 (Fig. 1). The number of people with access to safely managed service increased by 870 million for drinking water and by two billion for sanitation over the 20-year period. The percentage of the population receiving a basic or higher level of service (safely managed service, basic service, and at least basic service) increased from 75.7 to 89.1% for drinking water and from 54.8 to 76.9% for sanitation. Importantly, the number and proportion of the population receiving only a limited or unimproved level has also declined. These results show that global society is certainly advancing toward universal access to safely managed or basic services for drinking water and sanitation; however, more than 800 million people still live in serious conditions without basic WASH services. Additionally, access rates for basic and safely managed sanitation services are lower than those for drinking water, highlighting the need for further efforts, so as not to leave anyone behind, in terms of access to WASH services.

### Comparison of access rates with socioeconomic dimensions

Although a global statistics analysis showed that the number and proportion of people with access to improved services increased globally since 2000, additional analyses accounting for social changes are required to evaluate this progress accurately. For this purpose, we performed a regression analysis using JMP data as an objective variable and normalized socioeconomic data as an explanatory variable. We applied a mathematical model with logistic function for two reasons: (1) values of access rates, a model output, range from 0 to 1 and (2) access rates are expected to follow the law of diminishing marginal utility. The model includes a dummy variable (0 for the previous year, and 1 for the latest year, denoted as *C*_*d*_) to compare differences between years. The analyses were conducted for countries that have JMP and per capita GDP data, a known urban population ratio, a democracy index score, and per capita renewable internal freshwater resources explained on socioeconomic and political levels from 2000 to 2020. To account for the effects of inflation rates, we also performed analyses using GDP at purchasing power parity (GDP PPP), instead of per capita GDP, and obtained the same results, as shown below (Supplementary Tables 1–12).

**Fig. 2:**
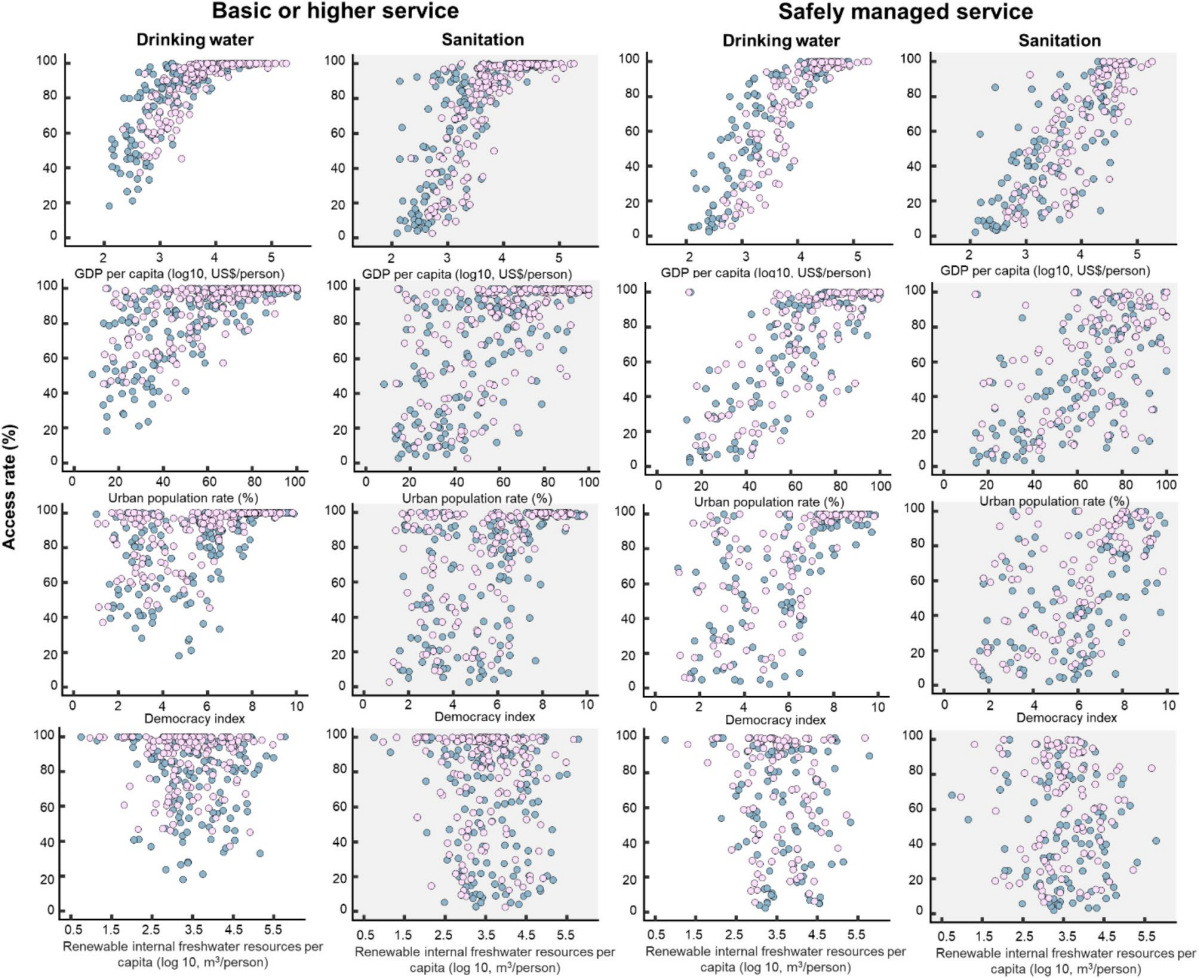
Association of access rates for drinking water and sanitation with socioeconomic, political, and hydrological statuses. Basic or higher level services represent the total access rate to safely managed service, basic service, and at least basic service. Dark blue and light pink plots denote data for 2000 and 2020, respectively. These figures describe the relationship between access rate and each indicator (i.e., GDP per capita, urban population rate, democracy index, and renewable internal freshwater resources per capita). The relationship is analyzed with a regression model as shown in Table 1 and Supplementary Table 13.

We investigated whether selected factors are associated with access rates for drinking water and sanitation using individual factor data as an explanatory variable (Fig. 2; Table 1). Among the used factors, per capita GDP, the urban population rate, and democracy index scores were identified as indicators significantly associated with the total percentage of the population with access to basic and safely managed services for drinking water and sanitation (i.e., percentage of people with a basic or higher level of service). This result is consistent with the analysis using only safely managed service data (Supplementary Table 13). Our finding also agrees with previous studies reporting the relationship of the access rate with socioeconomic and political dimenstions^5,7,13–15^. Based on this preliminary analysis, we decided to apply the per capita GDP, urban population rate, and democracy index score as explanatory variables for a multivariable model employing the logistic function in the following analysis.

We applied the multivariable model to compare access rate between 2000 and 2020 based on the same socioeconomical and political conditions (i.e., access rate adjusted for socioeconomical and political conditions). We found that countries with higher GDP per capita had higher access rates to all types of services. Countries with higher urban population rates tended to have higher access rates to services, except for safely managed sanitation services. Meanwhile, countries with higher democracy scores were positively correlated only with access to basic or higher levels of drinking water service (Table 2). We also observed dummy variable *C*_*d*_ for the basic or higher levels of sanitation service were significantly negative (−0.54, 95% credible interval (CrI): −0.86 to −0.22), which represents that the adjusted access rate in 2020 is lower than that in 2000. The value of *C*_*d*_ was significantly negative value (−0.74, 95%CrI: −1.07 to −0.42) for safely managed drinking service, but not considered significant negative for basic or higher levels of drinking water service (−0.18, 95%CrI: −0.40 to 0.04) and for safely managed sanitation service (−0.18, 95%CrI: −0.50 to 0.14). These results regarding *C*_*d*_ illustrate that the progress speed of improving access rate along with socioeconomical and political change differs between service type (i.e., drinking water vs. sanitation) and service levels (i.e., safely managed service vs. basic or higher levels of services).

We also performed the same multivariable analysis by using data on access rate to surface water (for drinking water service) and open defecation (for sanitation service) to explore the relationship between the worst service and socioeconomical and political factors (Supplementary Table 14). We found that countries with higher GDP per capita had lower access rates to the services. We also observed dummy variable *C*_*d*_ was significantly negative for surface water (−0.46, 95%CrI: −0.90 to −0.08), which represents that the adjusted access rates of the worst services in 2020 are lower than that in 2000. *C*_*d*_ for open defecation was − 0.26 (95%CrI: −0.68 to 0.12).

We also performed an analysis for the periods divided as the MDG era (i.e., 2000–2015) and SDG era (i.e., 2015–2020), since the definitions for service levels and access were updated in the SDG era^7^. When using data from the MDG era, all *C*_*d*_ values were significantly negative except for safely managed sanitation service (Supplementary Table 15). In contrast, *C*_*d*_ for the basic or higher levels of drinking water service was significantly positive during SDG era (Supplementary Table 16). The *C*_*d*_ values for the other services were neither significantly positive nor negative in SDG era. Overall, the adjusted access rates declined during the MDG era but were comparable or partially improved during the SDG era.

To examine different conditions between urban and rural areas, the same analysis was performed using data for 2000 and 2020, with information separating urban and rural areas. We firstly investigated the relationship between access rate and each indicator and observed a significant association between the access rates with per capita GDP, the urban population rate, and democracy index score for all data sets, as was seen in the combined data (Table 1, Supplementary Tables 13, 17–20). Further, per capita renewable internal freshwater resources were identified as an associated factor for safely managed drinking service in both rural and urban areas, and for safely managed sanitation in urban areas (Supplementary Tables 17–20). These results show the used indicators are potentially related to access rate in urban and rural area and so, we performed a regression analysis using all the socioeconomic, political, and hydrological factor data.

We found that per capita GDP is significantly positively associated with access rates for all types of services in both urban and rural areas (Supplementary Tables 21–22), representing that countries with higher GDP have higher access rate regardless of the areas. Interestingly, per capita renewable internal freshwater resources are significantly negatively associated with access rates, except for the basic or higher levels of sanitation service in urban areas and for safely managed sanitation in rural areas, illustrating that countries with higher per capita renewable water resources tended to have lower access rate. For urban areas, all values of *C*_*d*_ were significantly negative, regardless of service quality and type. For rural areas, values of *C*_*d*_ were not considered significantly negative for the basic or higher levels of drinking service (−0.14, 95%CrI: −0.41 to 0.12) and for safely managed sanitation service (−0.34, 95%CrI: −0.73 to 0.04), which is consistent with the results of the analysis that combines rural and urban area data (Table 2).

**Table 1:** Estimated parameters using data on basic or higher level services (Basic + at least basic + safely management).

Explanatory variable		Drinking water			Sanitation	
Variable	Intercept	*C*_*1*_	*C*_*d*_	Intercept	*C*_*1*_	*C*_*d*_
Per capita GDP	2.59 (2.37 to 2.82)	1.55 (1.40 to 1.72)	− 0.37 (− 0.56 to − 0.18)	1.91 (1.63 to 2.22)	1.96 (1.70 to 2.24)	− 0.60 (− 0.89 to − 0.33)
Urban population rate	1.87 (1.68 to 2.07)	1.00 (0.86 to 1.16)	0.48 (0.25 to 0.72)	1.01 (0.81 to 1.23)	1.09 (0.92 to 1.29)	0.34 (0.05 to 0.64)
Democracy index	1.43 (1.25 to 1.62)	0.60 (0.46 to 0.75)	0.68 (0.40 to 0.98)	0.64 (0.43 to 0.87)	0.67 (0.50 to 0.85)	0.62 (0.28 to 0.97)
Renewable water^a^	1.40 (1.21 to 1.59)	− 0.04 (− 0.19 to 0.10)	0.64 (0.30 to 1.00)	0.63 (0.42 to 0.86)	− 0.11 (− 0.28 to 0.05)	0.52 (0.18 to 0.88)

$$\:P\:=\frac{Exp(intercept+\:{C}_{1}{X}_{1}+{C}_{d}{X}_{d})}{1+\:Exp(intercept+\:{C}_{1}{X}_{1}+{C}_{d}{X}_{d})}$$.

^a^ Per capita renewable internal freshwater resources.

The values in parentheses represent a 95% Bayesian credible interval.

**Table 2:** Estimated parameters of a multivariable model employing the logistic function.

Service quality		Intercept	*C*_*1*_ (GDP)	*C*_*2*_ (UP^a^)	*C*_*3*_ (Democracy index)	*C*_*d*_
Basic or higher	Drinking water (150 countries)	2.33 (2.11 to 2.56)	1.07 (0.85 to 1.30)	0.37 (0.22 to 0.53)	0.16 (0.03 to 0.28)	− 0.18 (− 0.40 to 0.04)
	Sanitation (147 countries)	1.71 (1.42 to 2.03)	1.69 (1.35 to 2.06)	0.29 (0.07 to 0.51)	− 0.04 (− 0.24 to 0.16)	−0.54 (− 0.86 to − 0.22)
Safely managed	Drinking water (99 countries)	1.52 (1.26 to 1.81)	1.51 (1.18 to 1.87)	0.36 (0.14 to 0.59)	0.00 (− 0.19 to 0.18)	− 0.74 (− 1.07 to − 0.42)
	Sanitation (101 countries)	0.33 (0.11 to 0.55)	1.21 (0.88 to 1.56)	−0.07 (− 0.32 to 0.18)	0.04 (− 0.15 to 0.23)	− 0.18 (− 0.50 to 0.14)

$$P~ = \frac{{Exp\left( {intercept + C_{1} X_{1} \left( {GDP} \right) + C_{2} X_{2} \left( {Urban\;population} \right) + C_{3} X_{3} \left( {Democracy\;index} \right) + C_{d} X_{d} } \right)}}{{1 + Exp\left( {intercept + C_{1} X_{1} \left( {GDP} \right) + C_{2} X_{2} \left( {Urban\;population} \right) + C_{3} X_{3} \left( {Democracy\;index} \right) + C_{d} X_{d} } \right)}}$$.

^a^UP: Urban population rate.

The values in parentheses represent a 95% Bayesian credible interval.

### Association of socioeconomic conditions with improved access rate

In addition to the progress analyses and comparisons of access conditions, we analyzed the association of socioeconomic, political, and hydrological conditions with improved access rates from 2000 to 2020. This analysis was also performed using a multivariable model with a logistic function. To apply the regression model, we defined the attributable proportion of the recent improvement rate (i.e., (C_2020_–C_2000_)/C_2020_, where C_2000_ and C_2020_ represent crude access rates in 2000 and 2020, respectively) as an indicator for progress of improving accessibility. The indicator matches the concept of the regression model, in terms of its ranges (typically 0 to 1). A few countries showing an extraordinary decline in access rates between 2000 and 2020 were excluded from the analysis (see Methods); this was because negative value of improvement rate is out of range of the logistic model’s output. It should be noted that the number of removed countries were three and the impact of this procedure on the outcome is expected to be limited.

**Fig. 3:**
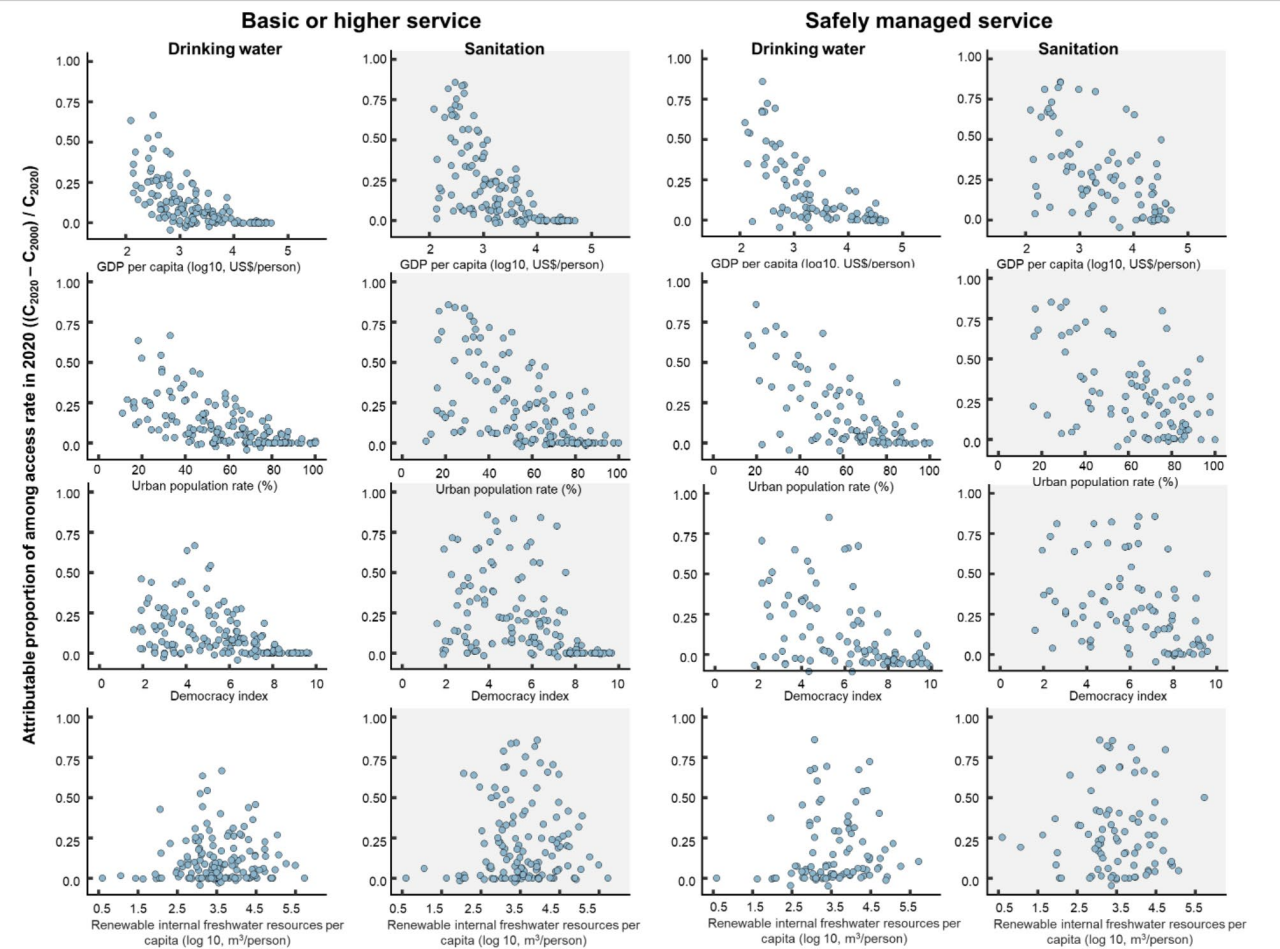
Association of improved access rates to drinking water and sanitation with socioeconomic, political, and hydrological status. Basic or higher level services represent the total access rate to safely managed service, basic service, and at least basic service. The vertical axis represents the contribution ratio of growing coverage between 2000 and 2020 to the access rate in 2020; for instance, 0.5 on the vertical axis means that half of the infrastructure in 2020 was constructed between 2000 and 2020. These figures describe the relationship between access rate and each indicator (i.e., GDP per capita, urban population rate, democracy index, and renewable internal freshwater resources per capita). The relationship is analyzed with a regression model as shown in Table 3.

The improved access rates for the basic or higher levels and safely managed services were negatively associated with GDP for both drinking water and sanitation (parameter *C*_*1*_), but were not necessarily associated with the other factors, except for the significantly positive association between a democracy index score and safely managed drinking water service (Fig. 3; Table 3). The *C*_*1*_ value shows that low-income countries tended to improve their access rates between 2000 and 2020, compared with high-income countries. Notably, lower income countries are likely to have lower access rates in 2000 than higher income countries, which were associated with higher improvement rates (Fig. 2; Table 1, Supplementary Fig. 1). Among those countries with the same income level, improved access rates were comparable regardless of socioeconomic, political, and hydrological conditions.

**Table 3:** Estimated parameters of a multivariable model employing the logistic function.

Service quality		Intercept	*C*_*1*_ (GDP)	*C*_*2*_ (UP^a^)	*C*_*3*_ (Democracy index)	*C*_*4*_ (RW^b^)
Basic or higher	Drinking water (143 countries)	− 2.48 (− 2.80 to − 2.21)	− 0.93 (− 1.33 to − 0.55)	− 0.19 (− 0.46 to 0.09)	− 0.03 (− 0.27 to 0.21)	0.11 (− 0.08 to 0.29)
	Sanitation (137 countries)	− 1.66 (− 1.98 to − 1.40)	− 1.25 (− 1.81 to − 0.77)	− 0.03 (− 0.37 to 0.33)	0.21 (− 0.10 to 0.54)	0.02 (− 0.21 to 0.24)
Safely managed	Drinking water (93 countries)	− 2.28 (− 2.83 to − 1.86)	− 1.67 (− 2.48 to − 0.99)	− 0.20 (− 0.66 to 0.24)	0.52 (0.15 to 0.95)	0.14 (− 0.15 to 0.43)
	Sanitation^c^ (86 countries)	− 1.12 (− 1.47 to − 0.83)	− 0.98 (− 1.75 to − 0.29)	− 0.08 (− 0.58 to 0.41)	0.33 (− 0.14 to 0.91)	− 0.03 (− 0.35 to 0.29)

$$P = \frac{{Exp\left( {intercept + C_{1} X_{1} \left( {GDP} \right) + C_{2} X_{2} \left( {Urban\;population} \right) + C_{3} X_{3} \left( {Democracy\;index} \right) + C_{4} X_{4} \left( {RW} \right)} \right)}}{{1 + Exp\left( {intercept + C_{1} X_{1} \left( {GDP} \right) + C_{2} X_{2} \left( {Urban\;population} \right) + C_{3} X_{3} \left( {Democracy\;index} \right) + C_{4} X_{4} \left( {RW} \right)} \right)}}$$.

^a^UP: Urban population rate.

^b^RW: Per capita renewable internal freshwater resources.

The values in parentheses represent a 95% Bayesian credible interval.

## Discussion

To enhance effective management and efforts toward achieving goals, such as universal access to safely managed WASH services, it is essential to perform retrospective analyses to provide feedback for program administrators and stakeholders. This study aimed to present helpful feedback on the progress toward SDG Targets 6.1 and 6.2, to realize one of the ultimate goals of human society, access to clear drinking water and sanitation for all, between 2000 and 2020. The uniqueness of the present study lies in evaluating global-scale progress while considering changes in socioeconomic conditions that have been identified as factors potentially associated with access rates for WASH services^5,7,13–16^. In addition, this study uses relatively recent SDG data (i.e., from 2000 to 2020) on both drinking water and sanitation to provide an updated and comprehensive view of the progress made to date.

We found that per capita GDP and the urban population rate, and democracy index score have significantly positive relationships with the access rates for drinking water and sanitation, while the hydrological factor was negatively associated with some access rates in rural and urban areas (Table 1, Supplementary Tables 13–20). The abundance of potential water resources available to drinking water and sanitation might act as a negative factor for the development of WASH services. For example, in regions where people have access to natural water sources (e.g., precipitations, rivers) and can manage with these resources, the government may have less incentive to invest in WASH services compared to countries where severe water shortages threaten lives and make WASH services essential for survival. However, the exact reasons for the impact of water resources on access rates remain unclear. Future research should be needed for revealing the relationship between access rates and hydrological factors as well as socioeconomic factors. The interaction between water-related access and socioeconomic development has been discussed in previous studies, suggesting that SDG Target 6 has a synergetic relationship with other SDG targets^19,20^. Our findings highlight the association between access rates and social status and the importance of considering countries’ socioeconomic and political status when analyzing their progress toward universal access to WASH services.

We discovered that access rates adjusted for socioeconomic conditions tended to be lower from 2000 to 2020, especially during MDG era (2000–2015), suggesting that the improvement of access rates has not progressed to the level expected based on the same socioeconomic and political indicators from 2000 to 2020. Although the definition of “access” from the MDG to SDG usage could be a reason for declining adjusted rate from 2000 to 2020, we did not support the possibility. This is because the adjusted access rates did not decline significantly between 2015 and 2020 in contrast to between 2000 and 2015. Notably, changes in the adjusted access rates between 2000 and 2020 vary depending on service type (i.e., drinking water and sanitation), service quality (i.e., safely managed service and basic or higher levels of service), and service areas (i.e., urban and rural). In urban regions, all adjusted access rates for drinking water and sanitation, regardless of service quality, have declined, presumably because the construction of WASH services’ infrastructures have not kept up with rapid population shifts to urban areas.

For rural areas, *C*_*d*_ values for sanitation were significantly negative for basic or higher levels of service, but were not significant for safely managed sanitation. This result suggests that some people have not gained access to basic services at a rate based on social development, while others did gain access to safely managed service along with social developments between 2000 and 2020. In other words, disparities in sanitation access might have grown in the past two decades. Interestingly, drinking water showed a trend different from that of sanitation. *C*_*d*_ values were significantly negative for safely managed drinking service, but were not significant for basic or higher levels of drinking water service. Countries may choose to provide at least basic drinking water service, rather than high quality service (i.e., safely managed service), due to financial limitations. For rural regions, upgrading from basic service to safely managed service is a crucial challenge ahead of 2030. Our results describe the different challenges related to the provision of drinking water and sanitation in rural areas; an awareness of these could be helpful when selecting appropriate measures for mitigating the current situation by combining other indicators.

Our overall results suggest that improvements in access rates have not advanced well in the past two decades, especially during the MDG era. Although national efforts and global cooperation have contributed to providing improved services for more than one billion people^4^, additional people could have been served if the access rates had improved along with rates of social development. This indication was contrary to our expectations, because recently developed technologies would seem to enable the provision of improved services of water-related infrastructures at a relatively lower cost than before. We assumed technology would lead to improvements in access rates, but the progress speed have slowed down probably due to decreased international development assistance for water-related projects in the past decade^21^ and decreased share of capital spending in the water sector^22^. Indeed, the share of capital spending is estimated to fall from 71.6% in 2009 to about 56.8% by 2020^22^. Previous reports have also stated that overall progress toward achieving the SDGs has not been fast enough, emphasizing the need of more effective international cooperation and management^14,21,22^. Coupling these claims with our findings, we strongly recommend reflecting ways of investment in water-related infrastructure and the management of WASH services, to attain the SDGs’ targets by 2030.

An analysis aimed at improving crude access rates showed that relatively low-income countries tended to improve their access rates for drinking water and sanitation more than high-income countries did between 2000 and 2020. This is reasonable because low-income countries had low access rate in 2000 and they had relatively more potential to increase these access rates than high-income countries. In our analysis, significant associations were found only for per capita GDP, while a previous study indicated that national socioeconomic characteristics might not be the primary determinants of progress in access to water and sanitiation^16^. It should also be noted that the modeling approach and data set used in this previous study were different from those used in our research; thus, its result cannot be simply compared with our results. Another previous study also suggested that ODA contributes to improving access rates in developing countries^14^, although our study did not include ODA in the model analysis, since the data were not available, or was “0” for most countries. Given that ODA accounts for approximately 6.9% of the total annual spending in the global water sector in 2017^22^, the impact ODA on the development of WASH infrastructure is not negligible. Further studies are required to warrant findings that can guide the efficient improvement of access rates using social, hydrological, and other related conditions.

The present study has several limitations. First, the JMP data might be biased since the data have been collected through household surveys and censuses in regions accessible to researchers. For example, higher data availability in urban areas than in rural areas could lead to both negatively and positively biased associations between WASH service access rates and the urban population rate. However, we confirmed that the urban population rate is positively correlated with access to all types of WASH services, suggesting minimal impact of data availability on the association (Tables 1 and 2). Also, other social indicators, apart from the four indicators used in this study, might be candidates for exploring the relationship with WASH service access rates. Using different WASH service data and social indicators could potentially reveal a novel association with access rates for WASH services; thus, further research is needed. Second, democracy index data for 2006 were used as the democracy index for 2000, which might have led to biased results. Finally, we could not prove causal relationships between social factors and access rates; thus, it should not be assumed that an improvement in socioeconomic status drives increased coverage rates. Notably, we investigated the temporal changes of access rates under fixed social conditions, suggesting the importance of economic conditions in improving access rates. Additional research is necessary to reveal the relationship between socioeconomic conditions and access rates for the effective management and progress toward universal access to WASH services. Despite these limitations, our results are meaningful for assessing the progress of SDG targets. The results demonstrated that access rates have improved, especially in relatively low-income countries, but they remain lower than they were in 2000, once adjustments are made for socioeconomic, political, and hydrological conditions. This leads to our recommendation to reflect past efforts and management plans, including investment, to achieve SDG targets by 2030.

## Materials and methods

### Data sets

We used JMP data on access rates for drinking water and sanitation from 2000 to 2020 to examine the progress toward universal access through a lens related to socioeconomic and hydrological conditions^12^. The JMP data were collected based on SDG indicators and have been used in previous studies^2,7^. The data were categorized on the basis of service quality (e.g., safely managed service, basic service, and at least basic service). The definitions of service quality levels are available on JMP websites^4^. Our analyses were performed using two categories of service quality: safely managed service and basic or higher service. Basic or higher service was an artificially created category representing an aggregation of safely managed service, basic service, and at least basic service. Also, we did not use hygiene data were due to its limited data size.

Based on previous findings, we selected four socioeconomic, political, and hydrological factors that are potentially associated with WASH service prevalence: per capita GDP, per capita GDP at purchasing power parity (GDP PPP), the urban population rate, democracy index score, and per capita renewable internal freshwater resources. The latter three factors were selected based on the following expectation: (1) higher urban population densities facilitate the efficient provision of WASH services, thereby increasing access rates, (2) countries with higher democracy indices are more likely to elect leaders focused on developing essential infrastructure for citizens, which is likely associated with greater access rates, (3) freshwater resources directly impact the availability of water, and affect the access rates. Finally, we expect freshwater resources directly affect available water resource and so related to the access rate. Based on these expectations, we selected these factors for the analysis. For the analyses, the data on per capita GDP, GDP PPP, and per capita renewable internal freshwater resources were transformed into logarithms. In addition, democracy index data for 2006 were used as data for 2000, because only data from 2006 were available. Socioeconomic and hydrological data were obtained from the World Bank^23–26^ and the Economist Intelligence Unit^27^. The datasets used are available on the GitHub repository (https://github.com/Hiroki-Ando1998/202406_SDG6_drinkingwater_sanitation_access).

### Data analysis

We first investigated a significant association between access rates with each socioeconomic, political, or hydrological factor. The significant associated factors were used as explanatory variables in a multivariable model in the subsequent analyses. These analyses were conducted for multiple combinations of datasets: 2000 and 2020, 2000 and 2015, 2015 and 2020, 2000 and 2020 in urban areas, and 2000 and 2020 in rural areas.

The first investigation was performed with the use of a model employing logistic function, a variable for a factor, and a dummy variable for years. This model analysis was conducted for countries that had both WASH data and each type of factor data. How representative the used data were was confirmed by comparing distribution shapes with complete datasets (Supplementary Figs. 2–4). Before the analysis, all factor data were normalized, as was also done in the other analyses explained below. Subsequently, using the significant associated factors as explanatory variables, a multivariable model analysis with logistic function was performed for countries with full coverage data and all factor data, as shown in Eq. (1)^16^.

1 $$P_{1} = \frac{{Exp\left( {intercept + C_{d} X_{d} + \sum C_{i} X_{i} } \right)}}{{1 + Exp\left( {intercept + C_{d} X_{d} + \sum C_{i} X_{i} } \right)}}$$

Where *P*_*1*_ is the access rate for drinking water or sanitation (-), *X*_*d*_ is dummy variable (0 for the previous year, and 1 for the later year), *C*_*d*_ represents a coefficient for the dummy variable, *X*_*i*_ represents factor data (e.g., per capita GDP), and *C*_*i*_ represents a coefficient for each parameter. The multicollinearity issue was not judged substantially influential, based on variance inflation factor values (Supplementary Table 23).

Finally, we examined the characteristics of countries that improved their access rates for drinking water and sanitation between 2000 and 2020 using an approach similar to that explained above. We used data that were not separated between urban and rural areas. In these analyses, improved access rates were defined as attributable proportions in access rates in 2020, as mathematically expressed in Eq. (2).

2 $$P_{2} = \frac{{C_{{2020}} - C_{{2000}} }}{{C_{{2020}} }}$$

Where *C*_*2000*_ and *C*_*2020*_ represent crude access rates for 2000 and 2020, respectively. The value of *P*_*2*_ generally ranged from 0 to 1, but several countries showed a value below 0. The countries with a *P*_*2*_ value lower than − 0.05 were excluded, with the consideration of measurement errors in the data aggregation process. The removed countries were as follows: (1) Burkina Faso, Central African Republic, and Zimbabwe for drinking water analyses and (2) Bosnia and Herzegovina, Democratic Republic of the Congo, and Zimbabwe for sanitation analyses.

The transformed variable was applied to a model employing logistic function, and all socioeconomic, political, and hydrological data were explanatory variables, as shown in Eq. (3). Although we investigated the association of each factor with the transformed variable, some parameters were not determined. Considering that each factor had a significant association with at least one access rate in the above analyses, we decided to use all factors’ data as explanatory variables. In the analyses, mean values of data from 2000 to 2020 were used to take into account a country’s conditions during the two intervening decades. A model analysis was performed for countries that had all data on the transformed variable and the factors from 2000 to 2020 (Supplementary Fig. 4).

3 $$P_{2} = \frac{{Exp\left( {intercept + \sum C_{i} X_{i} } \right)}}{{1 + Exp\left( {intercept + \sum C_{i} X_{i} } \right)}}$$

Where *P*_*2*_ is an attributable proportion (-), *X*_*i*_ represents mean factor data (e.g., per capita GDP), and *Ci* represents coefficients. The multicollinearity issue was not judged influential, based on variance inflation factor values (Supplementary Table 23).

Parameters (i.e., *C*_*i*_ and *C*_*d*_) were estimated using a Bayesian framework under the assumption that errors follow the normal distribution, as shown in Eq. (4).

4 $$C\sim norm\left( {P, sigma} \right)$$

Where C is JMP data on an access rate for drinking water or sanitation, *P* represents the estimated access rate (i.e., *P*_*1*_ or *P*_*2*_), and sigma is the standard deviation of normal distribution.

Parameter estimations were performed using the Markov chain Monte Carlo simulation under the conditions that simulations consisted of 10,000 iterations, 4 chains, and 2000 warm-ups. Prior distributions of all parameters were normal distribution, with a mean of 0 and standard deviation of 50. The estimated parameters were considered convergent when R-hat values of all parameters were less than 1.10. All of the computations were conducted in R-4.2.3 with a package {rstan}−2.21.2.

## Electronic supplementary material

Below is the link to the electronic supplementary material.

Supplementary Material 1

## Data Availability

The used data on access rate of drinking water, sanitation, GDP, the urban population rate, democracy index score, and per capita renewable internal freshwater resources are available on the GitHub repository (https://github.com/Hiroki-Ando1998/202406_SDG6_drinkingwater_sanitation_access).
