# Supplementary material for: Advancements in global water and sanitation access (2000–2020)
Source: Sci Rep. 2025 Feb 21;15:6399. doi: 10.1038/s41598-025-90980-7 (PMC11845473; doi:10.1038/s41598-025-90980-7)
Supplement: Supplementary file 1 — Supplementary Material 1 [file 41598_2025_90980_MOESM1_ESM.docx]

Supplementary material

Advancements in Global Water and Sanitation Access (2000-2020)

Hiroki Ando,^1,2^ Masaaki Kitajima,^1,3^ Taikan Oki,^4^ Michio Murakami^5^*

^1^ Division of Environmental Engineering, Faculty of Engineering, Hokkaido University, North 13 West 8, Kita-ku, Sapporo, Hokkaido 060-8628, Japan

^2^ Mel and Enid Zuckerman College of Public Health, University of Arizona, Tucson, AZ 85724, USA

^3^ Research Center for Water Environment Technology, Graduate School of Engineering, The University of Tokyo, 2-11-16 Yayoi, Bunkyo, Tokyo 113-0032, Japan

^4^ Department of Civil Engineering, Graduate School of Engineering, The University of Tokyo, 7-3-1 Hongo, Bunkyo, Tokyo 113-8656, Japan

^5^ Center for Infectious Disease Education and Research, Osaka University, 2-8 Yamadaoka, Suita, Osaka 565-0871, Japan

*Corresponding Author*

Michio Murakami*

Center for Infectious Disease Education and Research, Osaka University, 2−8 Yamadaoka, Suita, Osaka 565−0871, Japan

*Corresponding author’s email: michio@cider.osaka−u.ac.jp

**Supplemental Figures and Tables**

1. **Supplementary analysis with use of per capita GDP at purchasing power parity (GDP**_**PPP)**

**Table 1** Estimated parameters using data on basic or higher level services (Basic + at least basic + safely management) (corresponding to Table 2)

|  |  | Drinking water |  |  | Sanitation |  |
| --- | --- | --- | --- | --- | --- | --- |
|  | Intercept | C_1_ | C_d_ | Intercept | C_1_ | C_d_ |
| GDP_PPP | 2.31  (2.14–2.49) | 1.39  (1.26—–.52) | -0.15  (-0.32–0.02) | 1.70  (1.47–1.94) | 1.94  (1.72–2.18) | -0.42  (-0.67–-0.18) |

**Table 2** Estimated parameters using data on safely managed services between 2000 and 2020 (corresponding to supplementary Table 13)

|  |  | Drinking water |  |  | Sanitation |  |
| --- | --- | --- | --- | --- | --- | --- |
|  | Intercept | C_1_ | C_d_ | Intercept | C_1_ | C_d_ |
| GDP_PPP | 1.39  (1.19–1.59) | 1.78  (1.60–1.99) | -0.74  (-1.00–-0.50) | 0.30  (0.10–0.49) | 1.13  (0.96–1.31) | -0.19  (-0.47–0.09) |

**Table 3** Estimated parameters of a multivariable model employing the logistic function (corresponding to Table 2)

| Service quality |  | Intercept | C_1_ (GDP_PPP) | C_2_ (UP^a^) | C_3_ (Democracy index) | C_d_ |
| --- | --- | --- | --- | --- | --- | --- |
| Basic or higher | Drinking water  (147 countries) | 2.21  (2.03–2.39) | 1.05  (0.89–1.23) | 0.29  (0.15–0.44) | 0.17  (0.06–0.28) | -0.06  (-0.24–0.12) |
|  | Sanitation  (144 countries) | 1.62  (1.38–1.87) | 1.89  (1.58–2.24) | 0.11  (-0.10–0.32) | -0.04  (-0.22–0.14) | -0.44  (-0.72–-0.16) |
| Safely managed | Drinking water  (99 countries) | 1.33  (1.13–1.56) | 1.53  (1.23–1.85) | 0.20  (-0.01–0.42) | 0.04  (-0.14–0.22) | -0.64  (-0.94–-0.35) |
|  | Sanitation  (99 countries) | 0.31  (0.09–0.53) | 1.19  (0.86–1.54) | -0.09  (-0.36–0.18) | 0.12  (-0.08–0.31) | -0.16  (-0.49–0.16) |

**Table 4** Estimated parameters of a multivariable model employing the logistic function between 2000 and 2015 (corresponding to supplementary Table 14)

| Service quality |  | Intercept | C_1_ (GDP_PPP) | C_2_ (UP^a^) | C_3_ (Democracy index) | C_d_ |
| --- | --- | --- | --- | --- | --- | --- |
| Basic or higher | Drinking water  (150 countries) | 2.09  (1.92–2.28) | 0.95  (0.78–1.12) | 0.32  (0.16–0.47) | 0.18  (0.06–0.30) | -0.13  (-0.31–0.05) |
|  | Sanitation  (152 countries) | 1.46  (1.25–1.69) | 1.71  (1.43–2.02) | 0.11  (-0.09–0.32) | -0.02  (-0.20–0.14) | -0.42  (-0.68–-0.16) |
| Safely managed | Drinking water  (99 countries) | 1.34  (1.14–1.56) | 1.66  (1.35–2.00) | 0.07  (-0.16–0.29) | 0.00  (-0.18–0.16) | -0.62  (-0.91–-0.34) |
|  | Sanitation  (100 countries) | 0.22  (0.01–0.44) | 1.17  (0.84–1.52) | -0.04  (-0.32–0.23) | 0.18  (-0.02–0.36) | -0.14  (-0.46–0.18) |

**Table 5** Estimated parameters of a multivariable model employing the logistic function between 2015 and 2020 (corresponding to supplementary Table 15)

| Service quality |  | Intercept | C_1_ (GDP_PPP) | C_2_ (UP^a^) | C_3_ (Democracy index) | C_d_ |
| --- | --- | --- | --- | --- | --- | --- |
| Basic or higher | Drinking water  (153 countries) | 2.34  (2.19–2.51) | 0.97  (0.84–1.11) | 0.26  (0.15–0.38) | 0.18  (0.08–0.29) | 0.11  (-0.04–0.25) |
|  | Sanitation  (148 countries) | 1.65  (1.46–1.86) | 1.91  (1.67–2.18) | -0.08  (-0.25–0.09) | -0.11  (-0.28–0.05) | -0.01  (-0.23–-0.21) |
| Safely managed | Drinking water  (103 countries) | 1.28  (1.11–1.46) | 1.47  (1.22–1.74) | 0.16  (-0.03–0.34) | -0.05  (-0.18–0.12) | -0.11  (-0.34–0.11) |
|  | Sanitation  (100 countries) | 0.48  (0.29–0.67) | 1.30  (1.02–1.61) | -0.18  (-0.43–0.05) | 0.10  (-0.08–0.26) | -0.04  (-0.32–0.24) |

**Table 6** Estimated parameters using data on basic or higher level services in urban areas between 2000 and 2020 (corresponding to supplementary Table 16)

|  |  | Drinking water |  |  | Sanitation |  |
| --- | --- | --- | --- | --- | --- | --- |
|  | Intercept | C_1_ | C_d_ | Intercept | C_1_ | C_d_ |
| GDP _PPP | 3.21  (2.97–3.46) | 1.14  (1.00–1.30) | -0.25  (-0.47–-0.03) | 1.93  (1.71–2.16) | 1.58  (1.40–1.78) | -0.48  (-0.71–-0.25) |

**Table 7** Estimated parameters using data on safely managed services in urban areas between 2000 and 2020 (corresponding to supplementary Table 17)

|  |  | Drinking water |  |  | Sanitation |  |
| --- | --- | --- | --- | --- | --- | --- |
|  | Intercept | C_1_ | C_d_ | Intercept | C_1_ | C_d_ |
| GDP _PPP | 1.10  (0.89–1.32) | 1.27  (1.09–1.48) | -0.51  (-0.80–-0.24) | 0.18  (-0.01–0.39) | 1.18  (0.99–1.39) | -0.44  (-0.75–-0.14) |

**Table 8** Estimated parameters using data on basic or higher level services in rural areas between 2000 and 2020 (corresponding to supplementary Table 18)

|  |  | Drinking water |  |  | Sanitation |  |
| --- | --- | --- | --- | --- | --- | --- |
|  | Intercept | C_1_ | C_d_ | Intercept | C_1_ | C_d_ |
| GDP_PPP | 1.54  (1.35–1.75) | 1.25  (1.09–1.41) | -0.13  (-0.35–0.10) | 0.94  (0.72–1.17) | 1.74  (1.50–2.02) | -0.26  (-0.55–0.03) |

**Table 9** Estimated parameters using data on safely managed services in rural areas between 2000 and 2020 (corresponding to supplementary Table 19)

|  |  | Drinking water |  |  | Sanitation |  |
| --- | --- | --- | --- | --- | --- | --- |
|  | Intercept | C_1_ | C_d_ | Intercept | C_1_ | C_d_ |
| GDP_PPP | -0.46  (-0.69–-0.24) | 1.57  (1.32–1.85) | -0.52  (-0.87–-0.20) | -0.25  (-0.48–-0.02) | 1.17  (0.97–1.39) | -0.23  (-0.57–0.09) |

**Table 10** Estimated parameters of a multivariable model with data for urban areas between 2000 and 2020 (corresponding to supplementary Table 20)

| Service quality |  | Intercept | C_1_ (GDP_PPP) | C_2_ (UP^a^) | C_3_ (DI) | C_4_ (RW^b^) | C_d_ |
| --- | --- | --- | --- | --- | --- | --- | --- |
| Basic or higher | Drinking water  (139 countries) | 3.25  (2.98–3.55) | 1.08  (0.87–1.31) | 0.04  (-0.16–0.23) | 0.09  (-0.06–0.25) | -0.17  (-0.30–-0.04) | -0.30  (-0.54–-0.05) |
|  | Sanitation  (139 countries) | 1.88  (1.65–2.12) | 1.66  (1.40–1.96) | -0.01  (-0.20–0.18) | -0.13  (-0.31–0.03) | -0.02  (-0.15–0.11) | -0.54  (−0.81–-0.29) |
| Safely managed | Drinking water  (76 countries) | 1.12  (0.89–1.35) | 1.12  (0.85–1.41) | 0.19  (-0.03–0.42) | -0.02  (-0.21–0.15) | -0.25  (-0.86–-0.25) | -0.55  (−0.86–-0.25) |
|  | Sanitation  (97 countries) | 0.30  (0.07–0.53) | 1.48  (1.14–1.85) | -0.09  (-0.34–0.16) | -0.16  (-0.39–0.04) | -0.28  (-0.46–-0.12) | -0.65  (-1.02–-0.31) |

**Table 11** Estimated parameters of a multivariable model with data for rural areas between 2000 and 2020 (corresponding to supplementary Table 21)

| Service quality |  | Intercept | C_1_ (GDP_PPP) | C_2_ (UP^a^) | C_3_ (DI) | C_4_ (RW^b^) | C_d_ |
| --- | --- | --- | --- | --- | --- | --- | --- |
| Basic or higher | Drinking  (136 countries) | 1.45  (1.27–1.65) | 1.18  (0.95–1.42) | -0.12  (-0.30–0.06) | 0.28  (0.13–0.43) | -0.18  (-0.30–-0.06) | -0.03  (-0.27–0.21) |
|  | Sanitation  (135 countries) | 0.96  (0.73–1.21) | 1.98  (1.61–2.39) | -0.20  (-0.44–0.02) | 0.00  (-0.21–0.20) | -0.17  (-0.32–-0.02) | -0.36  (-0.68–-0.04) |
| Safely managed | Drinking  (64 countries) | -0.43  (-0.68–-0.19) | 1.82  (1.41–2.28) | -0.19  (-0.49–0.08) | 0.08  (-0.13–0.29) | -0.33  (-0.49–-0.18) | −0.58  (-0.97–-0.21) |
|  | Sanitation  (74 countries) | -0.22  (-0.48–0.03) | 1.29  (0.91–1.72) | -0.02  (-0.34–0.30) | -0.07  (-0.33–0.17) | 0.00  (-0.17–0.17) | -0.25  (-0.64–0.12) |

**Table 12** Estimated parameters of a multivariable model for improved rate employing the logistic function (corresponding to Table 3)

| Service quality |  | Intercept | C_1_ (GDP_PPP) | C_2_ (UP^a^) | C_3_ (Democracy index) | C_4_ (RW^b^) |
| --- | --- | --- | --- | --- | --- | --- |
| Basic or higher | Drinking water  (143 countries) | -2.36  (-2.64–-2.12) | -0.70  (-1.02–-0.41) | -0.23  (-0.49–0.04) | -0.10  (-0.33–0.14) | 0.07  (-0.11–0.27) |
|  | Sanitation  (134 countries) | -1.58  (-1.86–-1.34) | -1.05  (-1.51–-0.65) | -0.03  (-0.38–0.33) | 0.06  (-0.24–0.38) | 0.01  (-0.22–0.25) |
| Safely managed | Drinking water  (93 countries) | -2.06  (-2.49–-1.71) | -1.18  (-1.81–-0.64) | -0.31  (-0.80–0.16) | 0.42  (0.03–0.88) | 0.14  (-0.19–0.47) |
|  | Sanitation  (85 countries) | -1.14  (-1.50–-0.84) | -1.02  (-1.89–-0.36) | 0.01  (-0.56–0.59) | 0.32  (-0.15–0.92) | 0.01  (-0.31–0.33) |

^a^UP: Urban population rate

^b^RW: per capita renewable internal freshwater resources

The values in parentheses represent a 95% Bayesian credible interval.

1. **Supplementary analysis with use of per capita GDP**

**Table 13** Estimated parameters using data on safely management services between 2000 and 2020

|  |  | Drinking water |  |  | Sanitation |  |
| --- | --- | --- | --- | --- | --- | --- |
|  | Intercept | C_1_ | C_d_ | Intercept | C_1_ | C_d_ |
| Per capita GDP | 1.69  (1.44–1.97) | 1.86  (1.64–2.10) | -0.86  (-1.14–0.59) | 0.32  (0.12–0.53) | 1.14  (0.97–1.32) | -0.13  (-0.41–0.14) |
| Urban population rate | 1.00  (0.78–1.25) | 1.39  (1.17–1.64) | 0.10  (-0.20–0.42) | 0.06  (-0.16–0.27) | 0.78  (0.61–0.98) | 0.30  (-0.01–0.60) |
| Democracy index | 0.56  (0.32–0.82) | 0.84  (0.66–1.05) | 0.45  (0.08–0.82) | -0.09  (-0.32–0.14) | 0.69  (0.51–0.88) | 0.54  (0.22–0.88) |
| Renewable water^a^ | 0.53  (0.27–0.80) | −0.19  (-0.40–0.01) | 0.36  (-0.04–0.77) | -0.09  (-0.33–0.15) | -0.08  (-0.25–0.09) | 0.49  (0.15–0.85) |

$$P =\frac{Exp(intercept+ C_{1}X_{1}+C_{d}X_{d})}{1+ Exp(intercept+ C_{1}X_{1}+C_{d}X_{d})}$$

^a^Per capita renewable internal freshwater resources

The values in parentheses represent a 95% Bayesian credible interval.

**Table 14** Estimated parameters of a multivariable model employing the logistic function between 2000 and 2020

| Service quality |  | Intercept | C_1_ (GDP) | C_2_ (UP^a^) | C_3_ (Democracy index) | C_d_ |
| --- | --- | --- | --- | --- | --- | --- |
| Surface water | Drinking water  (146 countries) | -3.41  (-3.76–-3.11) | -0.72  (-1.17–-0.32) | -0.37  (-0.69–-0.07) | -0.04  (-0.22–0.14) | -0.46  (-0.90–-0.08) |
| Open defecation | Sanitation  (146 countries) | -2.57  (-2.96–-2.25) | -0.92  (-1.32–-0.54) | -0.38  (-0.66–-0.11) | 0.03  (-0.25–0.19) | -0.26  (-0.68–0.12) |

$$P =\frac{Exp(intercept+ C_{1}X_{1}(GDP)+C_{2}X_{2}(Urban population)+C_{3}X_{3}(Democracy index)+C_{d}X_{d})}{1+ Exp(intercept+ C_{1}X_{1}(GDP)+C_{2}X_{2}(Urban population)+C_{3}X_{3}(Democracy index)+C_{d}X_{d})}$$

^a^UP: Urban population rate

The values in parentheses represent a 95% Bayesian credible interval.

**Table 15** Estimated parameters of a multivariable model employing the logistic function between 2000 and 2015

| Service quality |  | Intercept | C_1_ (GDP) | C_2_ (UP^a^) | C_3_ (Democracy index) | C_d_ |
| --- | --- | --- | --- | --- | --- | --- |
| Basic or higher | Drinking water  (154 countries) | 2.21  (2.00–2.43) | 0.95  (0.73–1.18) | 0.40  (0.24–0.56) | 0.20  (0.08–0.32) | -0.28  (-0.49–0.07) |
|  | Sanitation  (156 countries) | 1.62  (1.35–1.91) | 1.57  (1.26–1.90) | 0.28  (0.07–0.50) | -0.03  (-0.20–0.14) | -0.61  (-0.91–0.31) |
| Safely managed | Drinking water  (99 countries) | 1.50  (1.24–1.79) | 1.49  (1.16–1.85) | 0.28  (0.05–0.52) | 0.02  (-0.16–0.19) | -0.72  (-1.05–-0.40) |
|  | Sanitation  (103 countries) | 0.26  (0.04–0.48) | 1.16  (0.83–1.51) | 0.00  (-0.26–0.26) | 0.09  (-0.10–0.27) | -0.19  (-0.52–0.12) |

$$P =\frac{Exp(intercept+ C_{1}X_{1}(GDP)+C_{2}X_{2}(Urban population)+C_{3}X_{3}(Democracy index)+C_{d}X_{d})}{1+ Exp(intercept+ C_{1}X_{1}(GDP)+C_{2}X_{2}(Urban population)+C_{3}X_{3}(Democracy index)+C_{d}X_{d})}$$

^a^UP: Urban population rate

The values in parentheses represent a 95% Bayesian credible interval.

**Table 16** Estimated parameters of a multivariable model employing the logistic function between 2015 and 2020

| Service quality |  | Intercept | C_1_ (GDP) | C_2_ (UP^a^) | C_3_ (Democracy index) | C_d_ |
| --- | --- | --- | --- | --- | --- | --- |
| Basic or higher | Drinking water  (155 countries) | 2.37  (2.19–2.56) | 0.97  (0.79–1.16) | 0.35  (0.21–0.49) | 0.17  (0.06–0.29) | 0.18  (0.01–0.34) |
|  | Sanitation  (150 countries) | 1.65  (1.41–1.90) | 1.77  (1.47–2.09) | 0.08  (-0.12–0.28) | -0.14  (-0.33–0.05) | 0.08  (-0.17–0.32) |
| Safely managed | Drinking water  (103 countries) | 1.36  (1.17–1.58) | 1.49  (1.20–1.80) | 0.22  (0.03–0.42) | -0.06  (-0.24–0.12) | -0.02  (-0.26–0.21) |
|  | Sanitation  (101 countries) | 0.49  (0.31–0.68) | 1.41  (1.11–1.73) | -0.26  (-0.50–-0.02) | 0.03  (-0.15–0.19) | 0.04  (-0.23–0.30) |

$$P =\frac{Exp(intercept+ C_{1}X_{1}(GDP)+C_{2}X_{2}(Urban population)+C_{3}X_{3}(Democracy index)+C_{d}X_{d})}{1+ Exp(intercept+ C_{1}X_{1}(GDP)+C_{2}X_{2}(Urban population)+C_{3}X_{3}(Democracy index)+C_{d}X_{d})}$$

^a^UP: Urban population rate

The values in parentheses represent a 95% Bayesian credible interval.

**Table 17** Estimated parameters using data on basic or higher level services in urban areas between 2000 and 2020

|  |  | Drinking water |  |  | Sanitation |  |
| --- | --- | --- | --- | --- | --- | --- |
|  | Intercept | C_1_ | C_d_ | Intercept | C_1_ | C_d_ |
| Per capita GDP | 3.33  (3.07–3.62) | 1.25  (1.07–1.43) | -0.42  (−0.65–-0.18) | 2.09  (1.83–2.38) | 1.63  (1.42–1.87) | -0.64  (-0.91– -0.38) |
| Urban population rate | 2.64  (2.43–2.86) | 0.75  (0.60–0.92) | 0.43  (0.15–0.72) | 1.30  (1.10–1.52) | 0.90  (0.74–1.08) | 0.24  (-0.03–0.52) |
| Democracy index | 2.49  (2.28–2.74) | 0.59  (0.44–0.76) | 0.45  (0.15–0.76) | 1.03  (0.83–1.26) | 0.56  (0.41–0.73) | 0.49  (0.16–0.82) |
| Renewable water^a^ | 2.37  (2.17–2.60) | 0.01  (-0.15–0.17) | 0.49  (0.12–0.91) | 0.99  (0.78–1.21) | -0.07  (-0.23–0.08) | 0.41  (0.08–0.76) |

$$P =\frac{Exp(intercept+ C_{1}X_{1}+C_{d}X_{d})}{1+ Exp(intercept+ C_{1}X_{1}+C_{d}X_{d})}$$

^a^Per capita renewable internal freshwater resources

The values in parentheses represent a 95% Bayesian credible interval.

**Table 18** Estimated parameters using data on safely managed services in urban areas between 2000 and 2020

|  |  | Drinking water |  |  | Sanitation |  |
| --- | --- | --- | --- | --- | --- | --- |
|  | Intercept | C_1_ | C_d_ | Intercept | C_1_ | C_d_ |
| Per capita GDP | 1.23  (0.98–1.50) | 1.31  (1.09–1.55) | -0.65  (-0.98–-0.34) | 0.23  (0.01–0.45) | 1.13  (0.94–1.34) | -0.41  (-0.72–-0.11) |
| Urban population rate | 0.74  (0.52–0.97) | 0.85  (0.67–1.06) | 0.05  (-0.27–0.37) | -0.06  (-0.27–0.16) | 0.79  (0.62–0.98) | 0.12  (-0.19–0.42) |
| Democracy index | 0.59  (0.35–0.84) | 0.53  (0.36–0.72) | 0.30  (-0.06–0.66) | -0.16  (-0.40–0.08) | 0.41  (0.24–0.60) | 0.36  (0.02–0.70) |
| Renewable water^a^ | 0.53  (0.28–0.79) | -0.21  (-0.40–-0.03) | 0.24  (-0.14–0.62) | -0.16  (-0.40–0.08) | -0.23  (-0.41–-0.06) | 0.32  (-0.02–0.66) |

$$P =\frac{Exp(intercept+ C_{1}X_{1}+C_{d}X_{d})}{1+ Exp(intercept+ C_{1}X_{1}+C_{d}X_{d})}$$

^a^Per capita renewable internal freshwater resources

The values in parentheses represent a 95% Bayesian credible interval.

**Table 19** Estimated parameters using data on basic or higher level services in rural areas between 2000 and 2020

|  |  | Drinking water |  |  | Sanitation |  |
| --- | --- | --- | --- | --- | --- | --- |
|  | Intercept | C_1_ | C_d_ | Intercept | C_1_ | C_d_ |
| Per capita GDP | 1.67  (1.46–1.90) | 1.33  (1.15–1.52) | -0.29  (-0.54–-0.06) | 1.00  (0.75–1.27) | 1.63  (1.38–1.92) | -0.36  (-0.67–-0.05) |
| Urban population rate | 1.05  (0.87–1.24) | 0.74  (0.59–0.89) | 0.47  (0.21–0.75) | 0.42  (0.20–0.64) | 0.90  (0.72–1.09) | 0.40  (0.07–0.73) |
| Democracy index | 0.85  (0.67–1.03) | 0.64  (0.50–0.79) | 0.71  (0.43–1.00) | 0.23  (0.00–0.46) | 0.69  (0.51–0.88) | 0.70  (0.35–1.06) |
| Renewable water^a^ | 0.84  (0.65–1.03) | -0.04  (-0.19–0.11) | 0.63  (0.31–0.96) | 0.26  (0.03–0.50) | -0.09  (-0.27–0.08) | 0.57  (0.22–0.94) |

$$P =\frac{Exp(intercept+ C_{1}X_{1}+C_{d}X_{d})}{1+ Exp(intercept+ C_{1}X_{1}+C_{d}X_{d})}$$

^a^Per capita Renewable internal freshwater resources

The values in parentheses represent a 95% Bayesian credible interval.

**Table 20** Estimated parameters using data on safely managed services in rural areas between 2000 and 2020

|  |  | Drinking water |  |  | Sanitation |  |
| --- | --- | --- | --- | --- | --- | --- |
|  | Intercept | C_1_ | C_d_ | Intercept | C_1_ | C_d_ |
| Per capita GDP | -0.35  (-0.61–-0.09) | 1.44  (1.19–1.74) | -0.56  (-0.94–-0.19) | -0.21  (-0.45–0.03) | 1.14  (0.94–1.36) | -0.25  (-0.59–0.08) |
| Urban population rate | -0.71  (-1.02–-0.42) | 0.95  (0.70–1.24) | 0.15  (-0.25–0.56) | -0.45  (-0.71–-0.20) | 0.83  (0.62–1.05) | 0.17  (-0.19–0.53) |
| Democracy index | -0.90  (-1.31–-0.54) | 0.86  (0.56–1.24) | 0.55  (0.11–1.01) | -0.66  (-0.99–-0.37) | 0.72  (0.49–0.98) | 0.53  (0.15–0.94) |
| Renewable water^a^ | -0.75  (-1.11–-0.42) | -0.41  (-0.68–-0.16) | 0.44  (-0.01–0.91) | -0.55  (0.86–0.25) | 0.02  (-0.19–0.24) | 0.48  (0.08–0.90) |

$$P =\frac{Exp(intercept+ C_{1}X_{1}+C_{d}X_{d})}{1+ Exp(intercept+ C_{1}X_{1}+C_{d}X_{d})}$$

^a^Per capita renewable internal freshwater resources

The values in parentheses represent a 95% Bayesian credible interval.

**Table 21** Estimated parameters of a multivariable model with data for urban areas between 2000 and 2020

| Service quality |  | Intercept | C_1_ (GDP) | C_2_ (UP^a^) | C_3_ (DI^b^) | C_4_ (RW^c^) | C_d_ |
| --- | --- | --- | --- | --- | --- | --- | --- |
| Basic or higher | Drinking water  (142 countries) | 3.32  (3.02–3.65) | 1.15  (0.87–1.45) | 0.02  (-0.17–0.22) | 0.12  (-0.04–0.28) | -0.18  (-0.31–-0.06) | -0.42  (-0.70–-0.14) |
|  | Sanitation  (142 countries) | 1.99  (1.71–2.30) | 1.58  (1.26–1.91) | 0.13  (-0.06–0.33) | -0.11  (−0.29–0.06) | -0.07  (-0.21–0.06) | −0.66  (-0.97–-0.36) |
| Safely managed | Drinking water  (76 countries) | 1.26  (1.01–1.54) | 1.22  (0.90–1.57) | 0.20  (-0.02–0.43) | -0.04  (−0.22–0.14) | -0.27  (-0.42–-0.12) | −0.73  (-1.08–-0.39) |
|  | Sanitation  (99 countries) | 0.31  (0.07–0.56) | 1.38  (1.03–1.77) | -0.02  (-0.26–0.21) | -0.23  (-0.47–0.00) | -0.25  (−0.42–-0.09) | -0.63  (-1.00–-0.28) |

$$P =\frac{Exp(intercept+ C_{1}X_{1}(GDP)+C_{2}X_{2}(Urban population)+C_{3}X_{3}(Democracy index)+C_{4}X_{4}\left( RW \right)+C_{d}X_{d})}{1+ Exp(intercept+ C_{1}X_{1}\left( GDP \right)+C_{2}X_{2}\left( Urban population \right)+C_{3}X_{3}\left( Democracy index \right)+C_{4}X_{4}\left( RW \right)+C_{d}X_{d})}$$

^a^UP: Urban population rate

^b^Democracy index

^c^Per capita renewable internal freshwater resources

The values in parentheses represent a 95% Bayesian credible interval.

**Table 22** Estimated parameters of a multivariable model with data for rural areas between 2000 and 2020

| Service quality | |  | | Intercept | | C_1_ (GDP) | | C_2_ (UP^a^) | | C_3_ (DI^b^) | | C_4_ (RW^c^) | | C_d_ | |
| --- | --- | --- | --- | --- | --- | --- | --- | --- | --- | --- | --- | --- | --- | --- | --- |
| Basic or higher | | Drinking  (139 countries) | | 1.55  (1.33–1.79) | | 1.16  (0.89–1.44) | | -0.01  (-0.19–0.17) | | 0.25  (0.08–0.41) | | -0.19  (-0.32–-0.08) | | -0.14  (-0.41–0.12) | |
|  | Sanitation  (138 countries) | | 1.04  (0.77–1.34) | | 1.79  (1.41–2.24) | | -0.07  (-0.32–0.16) | | -0.04  (-0.26–0.18) | | -0.16  (-0.32–-0.01) | | -0.44  (-0.81–-0.10) | |  |
| Safely managed | | Drinking  (64 countries) | | -0.24  (-0.53–0.04) | | 1.77  (1.32–2.29) | | -0.06  (-0.34–0.22) | | -0.03  (-0.27–0.21) | | -0.36  (-0.55–-0.19) | | -0.75  (-1.19–-0.32) | |
|  | | Sanitation  (74 countries) | | -0.15  (-0.42–0.12) | | 1.34  (0.94–1.78) | | -0.06  (-0.37–0.23) | | -0.11  (-0.37–0.13) | | 0.04  (-0.13–0.21) | | -0.34  (-0.73–0.04) | |

$$P =\frac{Exp(intercept+ C_{1}X_{1}(GDP)+C_{2}X_{2}(Urban population)+C_{3}X_{3}(Democracy index)+C_{4}X_{4}\left( RW \right)+C_{d}X_{d})}{1+ Exp(intercept+ C_{1}X_{1}\left( GDP \right)+C_{2}X_{2}\left( Urban population \right)+C_{3}X_{3}\left( Democracy index \right)+C_{4}X_{4}\left( RW \right)+C_{d}X_{d})}$$

^a^UP: Urban population rate

^b^Democracy index

^c^Per capita renewable internal freshwater resources

The values in parentheses represent a 95% Bayesian credible interval.

**
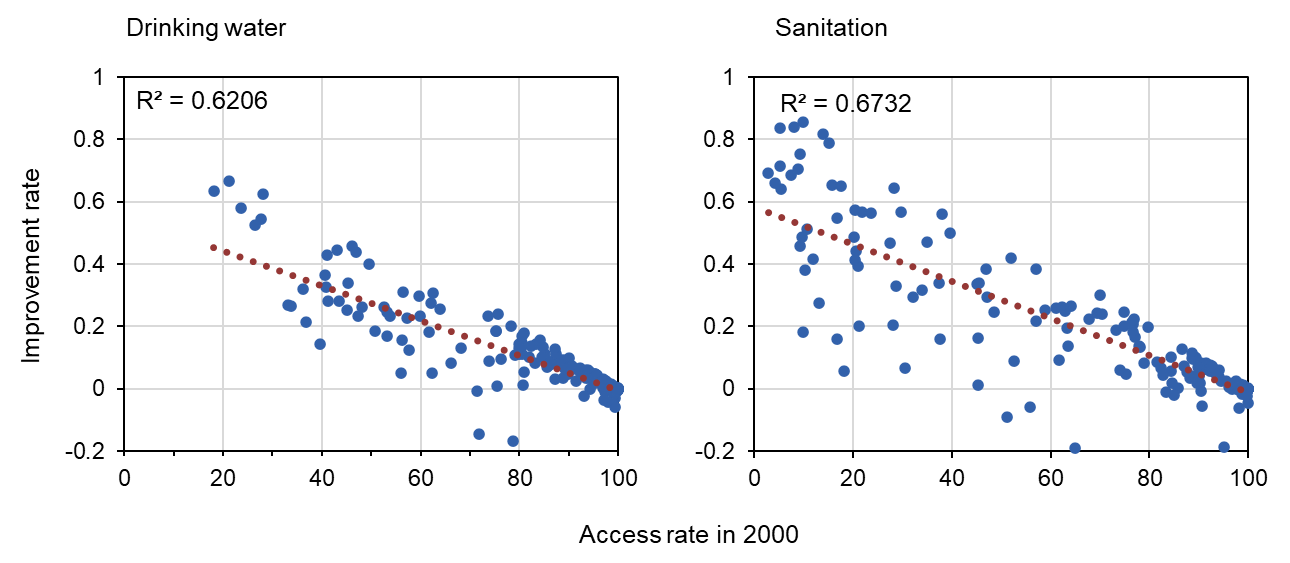
**

**Figure 1** Relationship between access rate in 2000 and improvement rate from 2000 to 2020. These figures describe the lower accee rates to basic or higer WASH services in 2009 tend to achive highber improvement rate of the access rates during the two decades.

**
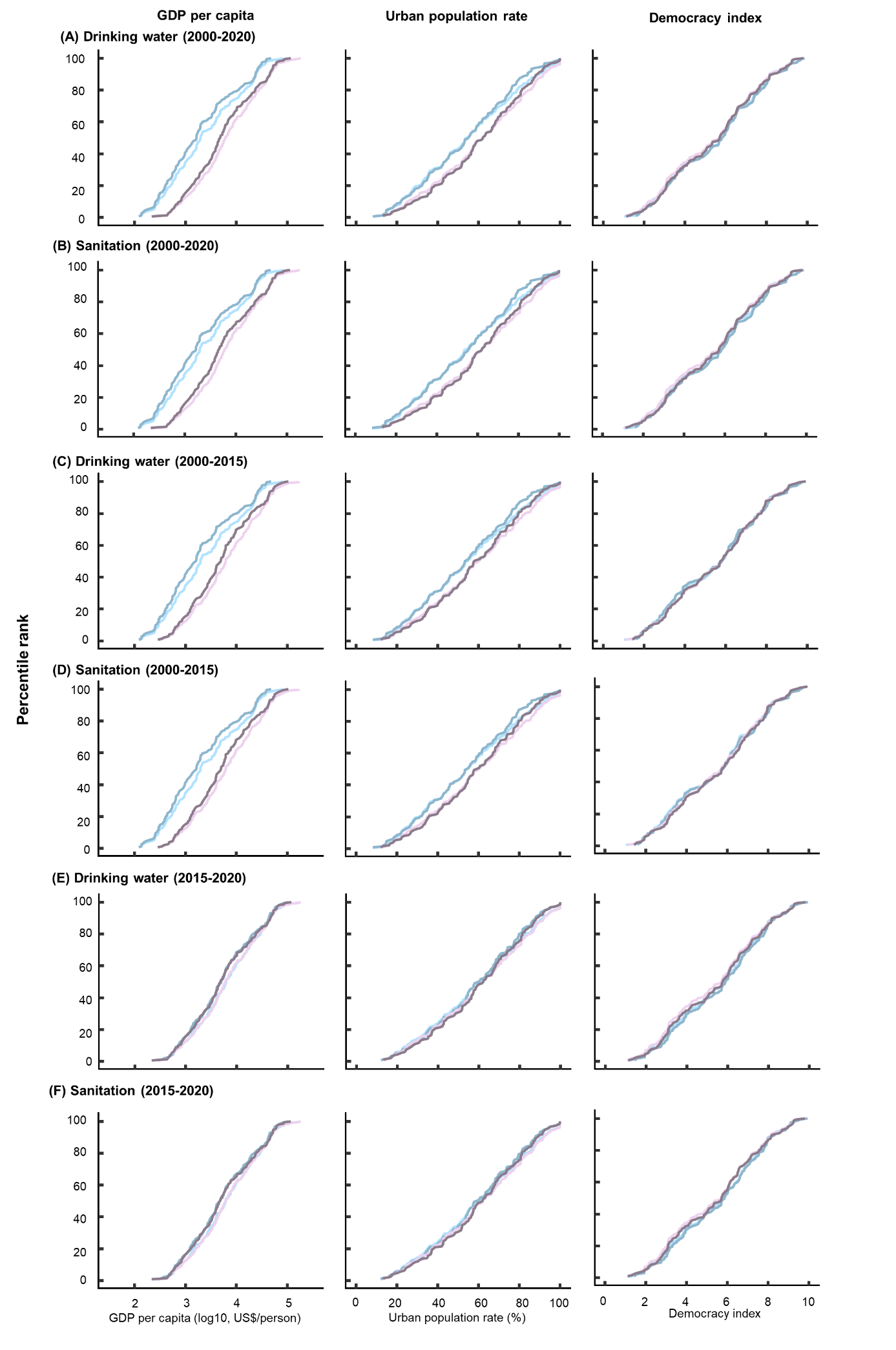
**

**Figure 2** Distribution of percentile ranks of socioeconomic and political indicators. Thicker colored lines denote the distribution of all indicator data we were able to collect, while lighter colored lines show the distribution of indicator data used for a multivariable model analysis of access rates for basic or higher level drinking or sanitation services (Tables 2, S2, S3). Blue and pink lines represent the data for the previous year and later years, respectively. (A) Drinking water access rate from 2000 to 2020, (B) Sanitation access rate from 2000 to 2020, (C) Drinking water access rate from 2000 to 2015, (D) Sanitation access rate from 2000 to 2015, (E) Drinking water access rate from 2015 to 2020, (F) Sanitation access rate from 2015 to 2020.

**
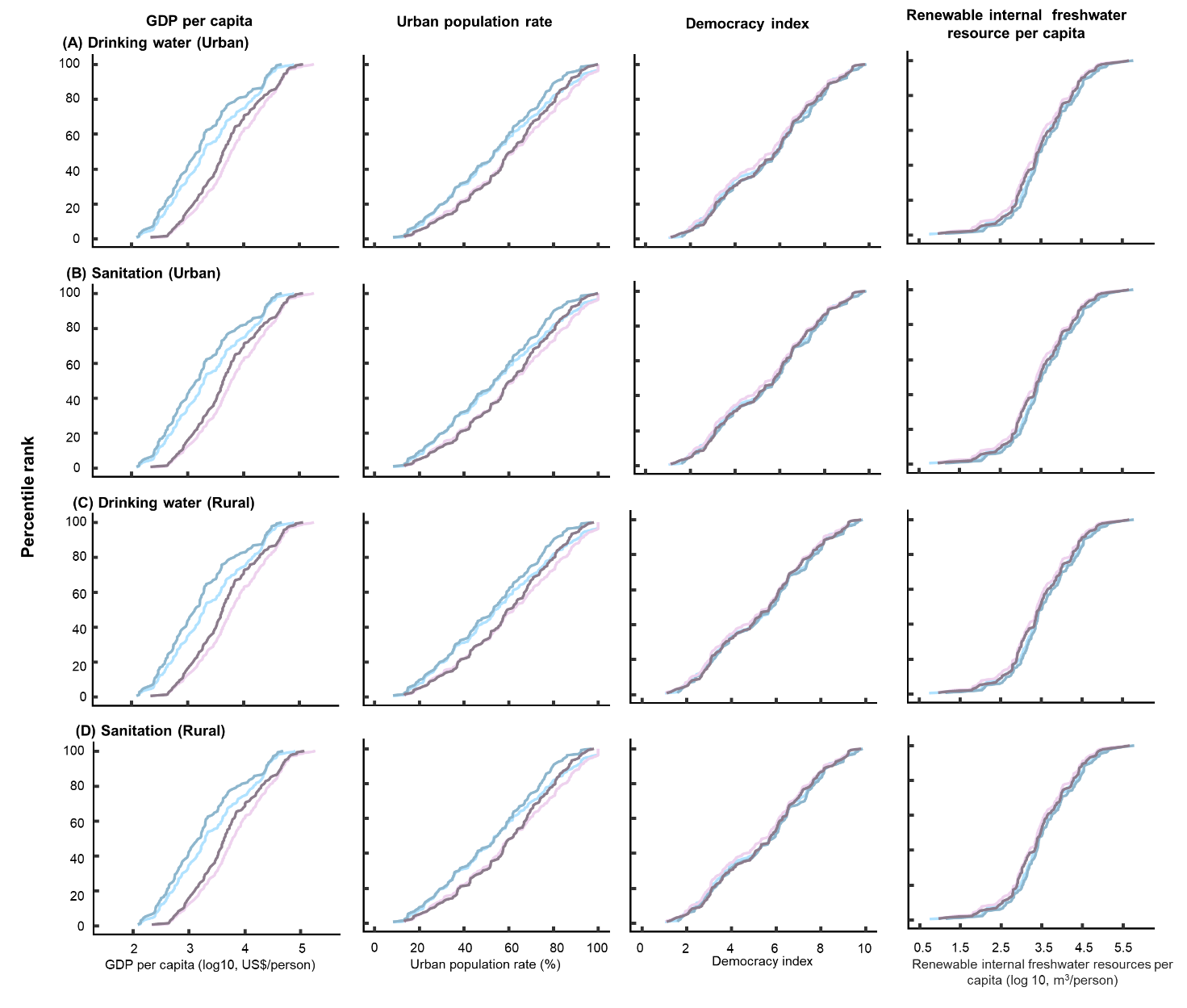
**

**Figure 3** Distribution of percentile ranks of socioeconomic and political indicators. Thicker colored lines denote the distribution of all indicator data we were able to collect, while lighter colored lines show the distribution of indicator data used for a multivariable model analysis of access rates for basic or higher level drinking or sanitation services (Tables S8, S9). Blue and pink lines represent the data for the years 2000 and 2020, respectively. (A) Drinking water access rate in urban areas, (B) Sanitation access rate in urban areas, (C) Drinking water access rate in rural areas, (D) Sanitation access rate in rural areas.


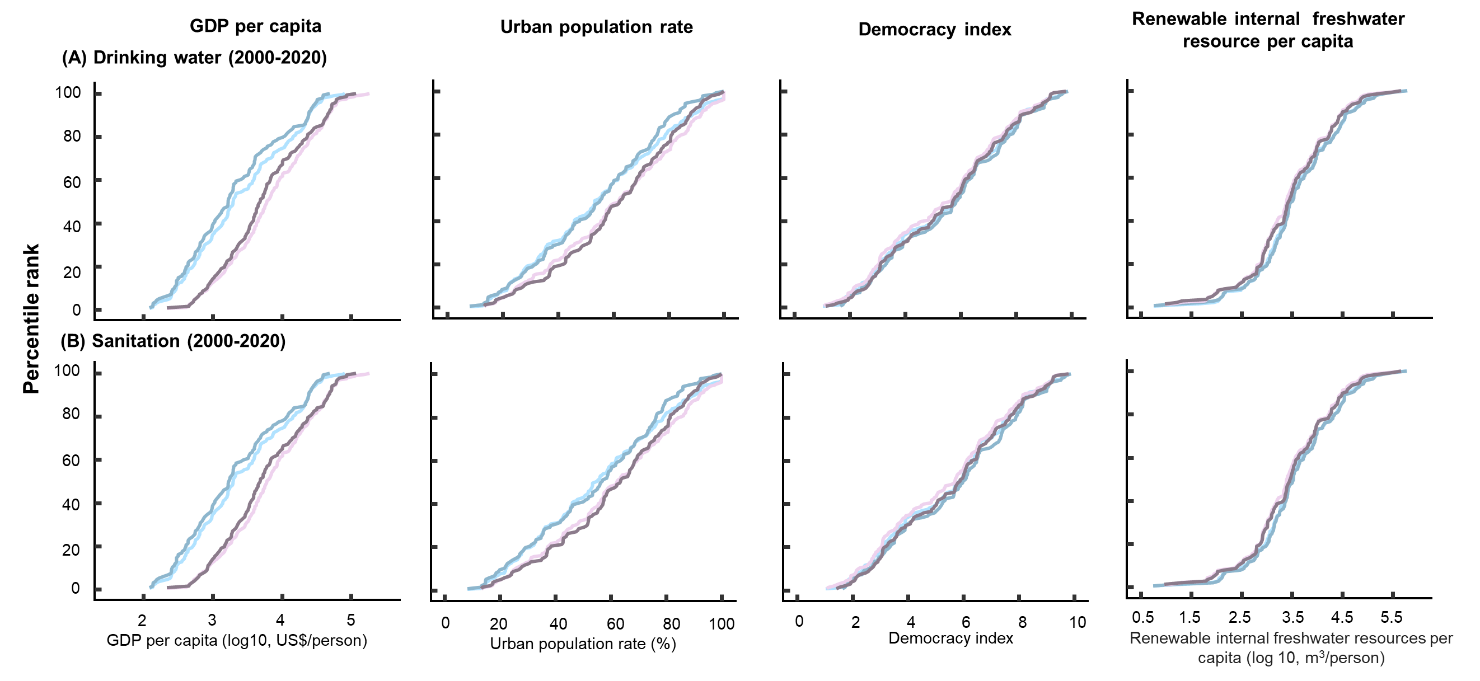


**Figure 4** Distribution of percentile ranks of socioeconomic and political indicators. Thicker colored lines denote the distribution of all indicator data we were able to collect, while lighter colored lines show the distribution of indicator data used for a multivariable model analysis for improved access rates for basic or higher level drinking or sanitation services from 2000 to 2020 (Table 3). Blue and pink lines represent the data for the years 2000 and 2020, respectively. (A) Drinking water access rate, (B) Sanitation access rate.

**Table 23** Variance inflation factor (VIF)

|  | Service quality | Service type | C_1_ (GDP^a^) | C_2_ (UP^b^) | C_3_ (DI^c^) | C_4_ (RW^d^) | C_d_ |
| --- | --- | --- | --- | --- | --- | --- | --- |
| Table 2 | Basic or higher | Drinking water | 4.35 | 2.92 | 1.71 | - | 1.27 |
|  |  | Sanitation | 4.62 | 2.99 | 1.85 | - | 1.29 |
|  | Safely managed | Drinking water | 5.31 | 3.15 | 2.19 | - | 1.35 |
|  |  | Sanitation | 4.99 | 3.18 | 1.88 | - | 1.28 |
| Table 3 | Basic or higher | Drinking water | 4.45 | 3.11 | 2.06 | 1.21 | - |
|  |  | Sanitation | 4.73 | 3.23 | 2.18 | 1.19 | - |
|  | Safely managed | Drinking water | 5.61 | 3.36 | 2.85 | 1.23 | - |
|  |  | Sanitation | 5.57 | 3.40 | 2.86 | 1.33 | - |
| Table S2 | Basic or higher | Drinking water | 4.44 | 3.12 | 1.62 | - | 1.26 |
|  |  | Sanitation | 4.63 | 3.13 | 1.73 | - | 1.27 |
|  | Safely managed | Drinking water | 5.42 | 3.35 | 2.09 | - | 1.33 |
|  |  | Sanitation | 5.19 | 3.38 | 1.86 | - | 1.26 |
| Table S3 | Basic or higher | Drinking water | 3.79 | 2.67 | 1.78 | - | 1.01 |
|  |  | Sanitation | 3.87 | 2.70 | 1.82 | - | 1.01 |
|  | Safely managed | Drinking water | 4.73 | 2.94 | 2.26 | - | 1.00 |
|  |  | Sanitation | 4.24 | 3.03 | 1.75 | - | 1.01 |
| Table S8 | Basic or higher | Drinking water | 4.63 | 2.78 | 2.23 | 1.07 | 1.32 |
|  |  | Sanitation | 4.43 | 2.75 | 2.09 | 1.09 | 1.30 |
|  | Safely managed | Drinking water | 4.63 | 3.08 | 2.09 | 1.06 | 1.36 |
|  |  | Sanitation | 4.20 | 2.63 | 1.97 | 1.14 | 1.34 |
| Table S9 | Basic or higher | Drinking water | 4.42 | 2.74 | 2.09 | 1.09 | 1.31 |
|  |  | Sanitation | 4.36 | 2.74 | 2.09 | 1.09 | 1.28 |
|  | Safely managed | Drinking water | 4.79 | 2.89 | 1.93 | 1.05 | 1.45 |
|  |  | Sanitation | 5.05 | 3.16 | 2.12 | 1.08 | 1.32 |

^a^ Per capita GDP

^b^ Urban population rate

^c^Democracy index

^d^Per capita renewable internal freshwater resources
